# Successful cold polypectomy for ectopic endometriosis in a narrow distal bile duct using a new slim cholangioscope

**DOI:** 10.1055/a-2598-4309

**Published:** 2025-05-26

**Authors:** Hirotsugu Maruyama, Yuki Ishikawa-Kakiya, Yuji Kawata, Tatsuya Kurokawa, Yoshinori Shimamoto, Kojiro Tanoue, Yasuhiro Fujiwara

**Affiliations:** 1 Department of Gastroenterology, Graduate School of Medicine, Osaka Metropolitan University, Osaka, Japan

Polyps in the bile duct are rare 1 2 . Most cases have been treated surgically, and there are few reports of polypectomy without concomitant surgery 3 4 5 . We report the first case of successful cold polypectomy in the hilar bile duct using a new slim cholangioscope (DRES Slim Scope and CMOS Camera; Japan Lifeline. Co., Ltd, Tokyo, Japan).

A 66-year-old woman was referred for evaluation of jaundice. Endoscopic retrograde cholangiopancreatography was performed, but the distal bile duct was narrow and a conventional cholangioscope could not be inserted into the bile duct. An inflammatory polyp was diagnosed by biopsy. Contrast-enhanced computed tomography confirmed the absence of blood flow to the polyp ( Fig. 1 ). We attempted polypectomy using a new slim cholangioscope ( Video 1 ) because the polyp was located in the hepatic hilar and surgery would have been excessive.

**Fig. 1:**
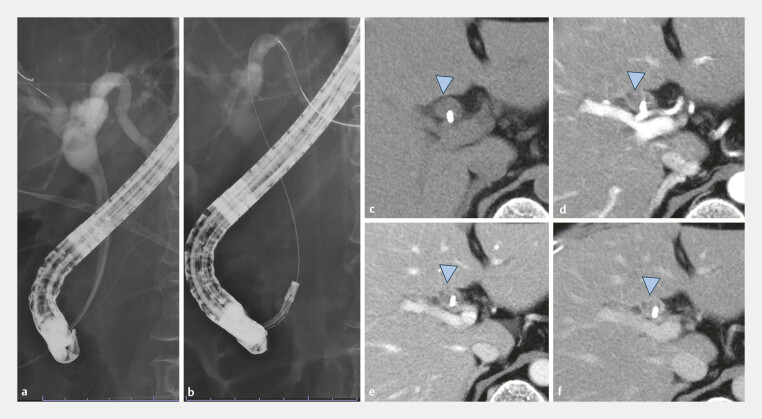
Fluoroscopy ( **a ,b** ) and preoperative computed tomography findings ( **c–f** ). **a** Narrow distal bile duct. **b** A conventional cholangioscope could not be inserted into the bile duct. **c–f** We confirmed the absence of blood flow to the polyp. **c** The polyp in the bile duct (blue arrowhead). **d** Arterial phase. **e** Portal phase. **f** Delay phase.

Cold polypectomy with a new slim cholangioscope.Video 1

The slim cholangioscope was inserted into the bile duct, the polyp was located, and the absence of tumor vessels was confirmed ( Fig. 2 ). After removing the slim cholangioscope, the snare (SpyGlass retrieval snare; Boston Scientific, Marlborough, Massachusetts, USA) was advanced over the guidewire to the hepatic hilar. Next, a 5-Fr cytology brush outer sheath was inserted into the bile duct.

**Fig. 2:**
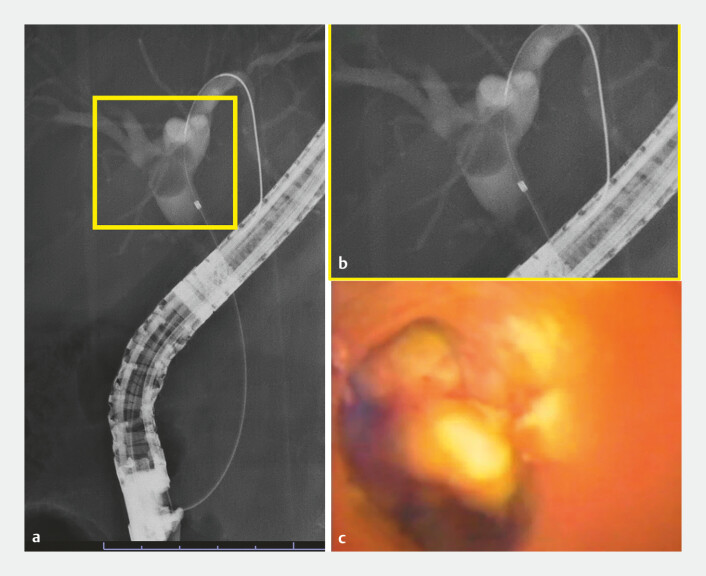
The polyp on imaging. **a, b** Fluoroscopy revealed the polyp (yellow frame shows enlarged view). **c** We confirmed the absence of tumor vessels using the slim cholangioscope.

A complementary metal oxide semiconductor (CMOS) camera was then passed through the outer sheath to observe the polyp ( Fig. 3 ). Finally, we performed cold polypectomy using a snare under direct visualization via the CMOS camera.

**Fig. 3:**
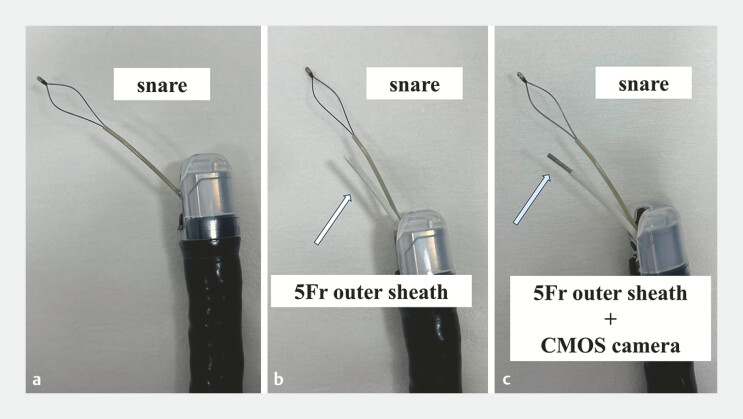
Polypectomy procedure. **a** The snare was inserted into the bile duct over the guidewire. **b** A 5-Fr cytology brush outer sheath was inserted into the bile duct while leaving the guidewire in the bile duct. **c** Then, the complementary metal oxide semiconductor (CMOS) camera was advanced into the outer sheath and polypectomy was performed under direct visualization.

After endoscopic treatment, the patient was discharged without any adverse events and progressed without recurrence. Stromal tissue similar to endometrial stroma was revealed. Immunostaining was positive for estrogen receptors, leading to a diagnosis of ectopic endometriosis of the bile duct ( Fig. 4 ).

**Fig. 4:**
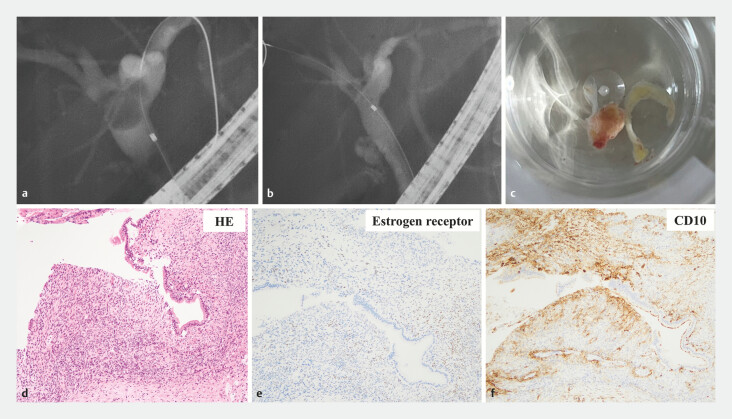
Polypectomy in the hilar biliary duct and histopathological findings. **a** Fluoroscopic image before endoscopic treatment. **b** The defect in the hilar biliary duct disappeared after endoscopic treatment. **c** The polyp after polypectomy. **d** Hematoxylin and eosin stain. **e, f** The sample was positive for estrogen receptor stain ( **e** ) and CD10 ( **f** ). Stromal tissue that was similar to endometrial stroma was observed.

The reusable CMOS camera facilitates procedures such as polypectomy. This method also enables the use of an electrochemical snare and represents a new advancement in future biliary treatment strategies.

Endoscopy_UCTN_Code_TTT_1AR_2AB

## Notes

Endoscopy E-Videos https://eref.thieme.de/e-videos
*E-Videos* is an open access online section of the journal *Endoscopy* , reporting on interesting cases and new techniques in gastroenterological endoscopy. All papers include a high-quality video and are published with a Creative Commons CC-BY license. Endoscopy E-Videos qualify for HINARI discounts and waivers and eligibility is automatically checked during the submission process. We grant 100% waivers to articles whose corresponding authors are based in Group A countries and 50% waivers to those who are based in Group B countries as classified by Research4Life (see: https://www.research4life.org/access/eligibility/ ). This section has its own submission website at https://mc.manuscriptcentral.com/e-videos .
